# Exploring perceptions of health literacy, healthcare access, and utilisation among higher education students in Alentejo, Southern Portugal: A qualitative study

**DOI:** 10.1371/journal.pone.0326575

**Published:** 2025-06-27

**Authors:** Jorge Rosário, Joana Correia Pires, Sónia Dias, Ana Rita Pedro

**Affiliations:** 1 Polytechnic Institute of Beja, Beja, Portugal; 2 Institute for Research and Advanced Training, University of Évora, Évora, Portugal; 3 Comprehensive Health Research Centre, CHRC, University of Évora, Évora, Portugal; 4 NOVA National School of Public Health, Public Health Research Centre, Comprehensive Health Research Center, CHRC, NOVA University Lisbon, Lisbon, Portugal; 5 NOVA National School of Public Health, Public Health Research Centre, Comprehensive Health Research Center, CHRC, REAL, CCAL, NOVA University Lisbon, Lisbon, Portugal; 6 NOVA National School of Public Health, Public Health Research Centre, Comprehensive Health Research Center, CHRC, REAL, CCAL, NOVA University Lisbon, Lisbon, Portugal; Ataturk University, Faculty of Pharmacy, TÜRKIYE

## Abstract

**Introduction:**

Health Literacy (HL), defined as the ability to access, understand, appraise and apply health information to make informed decisions, is essential for individual and public health. However, in rural regions such as Alentejo, Portugal, higher education students often encounter specific challenges that hinder the development and effective use of these skills. This study aimed to explore the perceptions of higher education students in Alentejo, Portugal, with regard to health literacy, access to and utilisation of healthcare, and to identify barriers, facilitators and interventions to inform strategies that promote equity, build student capacity and sustainably improve health outcomes.

**Methodology:**

A qualitative, exploratory, and descriptive study was conducted using three focus groups held on 11^th^, 13^th^, and 18^th^ December 2023, with a purposive non-probability sample of 29 participants across first-year, intermediate, and final-year levels. The participants shared their experiences, challenges, and resources related to health literacy, healthcare access, and utilisation. The focus group discussions were audio-recorded, transcribed verbatim, and analysed through a systematic process of open, axial, and selective coding. Codes were organised into categories, and thematic identification was facilitated using a constant comparative method. Content analysis was conducted with MAXQDA® 22 software. Ethical approval was obtained from the Ethics Committee of the University of Évora, Portugal, and informed consent was secured from all participants.

**Results:**

The students’ perception was that health literacy, access to and utilisation of healthcare are influenced by a dynamic interaction of barriers and facilitators. The identified barriers encompassed the presence of misinformation, financial and logistical challenges, and emotional stigma, particularly in the context of mental and sexual health. Conversely, facilitators were identified as encompassing access to digital tools, a family and peer support network, professional guidance and institutional resources such as libraries and workshops. The students recommended that interventions to improve health literacy be integrated into the higher education curriculum, that navigation support systems be improved and that structural inequalities be explored in order to improve access to and participation in healthcare.

**Conclusion:**

Digital resources have the potential to improve access to health information; however, persistent challenges such as misinformation, financial constraints and stigma limit their impact. This study underscores the significance of specific interventions to enhance health literacy and healthcare navigation skills. These interventions include the integration of health literacy into academic curricula, the provision of specialised workshops, and the facilitation of access to healthcare services. The study identifies nursing interventions and institutional support as key strategies to promote equitable access to healthcare and improve student wellbeing.

## Introduction

Health Literacy (HL) encompasses the knowledge, motivation, and competency required to access, understand, appraise, and apply health information in order to make judgements and take decisions in everyday life about healthcare, disease prevention, and health promotion [1].

As a modifiable determinant of health, it integrates public health and individual-level approaches and is embedded in health, social, and educational policies [2–7]. The correlation between HL levels and improved health outcomes, reduced inequalities, and decreased economic burden is well-established [8–11].

HL has proven to be a more effective predictor of health status than many socioeconomic factors [12] and is recognised as a critical determinant within health systems [13]. Conversely, low HL has been associated with an elevated mortality risk [14,15], increased utilisation of emergency and primary care services, reduced uptake of preventive care, and diminished quality of life [16–18].

In rural and sparsely populated regions such as Alentejo in Portugal, a lack of health resources, geographical isolation and economic challenges exacerbate existing inequalities [19–23]. The region is also characterised by low population density and high rates of student attrition, which exacerbate the structural barriers faced by higher education students [22,24].

These students encounter specific difficulties in navigating healthcare systems [24–26], difficulties that are exacerbated by systemic limitations, including a persistent shortage of healthcare services and constraints imposed by geographical remoteness [19,26,27]. Consequently, this demographic is a key focus in investigations into barriers and facilitators to health literacy [24].

The Integrated Model of Health Literacy is a widely applied model in European research [6,28–32]. It provides a structured framework for understanding how health literacy influences behaviours and outcomes, outlining key determinants (antecedents) and their effects on health outcomes (consequences) [5].

This model defines four core competencies of health literacy: access (the ability to seek and obtain health information), understand (the ability to comprehend information), appraise (the ability to interpret, evaluate, and filter information), and apply (the ability to use information to make informed health decisions).

These competencies are essential across the health continuum, covering healthcare, disease prevention, and health promotion [5]. Their effectiveness is shaped by organisational structures and resource availability, which influence individuals’ capacity to make informed decisions for themselves and those around them [33].

Within this broader framework, Navigational Health Literacy (NHL), an essential subset of health literacy, has gained prominence due to the increasing complexity of modern healthcare systems. NHL refers to the ability to navigate fragmented healthcare environments, make informed decisions regarding service utilisation, and engage with healthcare providers effectively [11,34]. NHL is defined as the knowledge, motivation, and skills individuals possess to access, understand, appraise, and apply information and communication in various forms, which are necessary to navigate healthcare systems and services effectively in order to obtain the most appropriate care for themselves or others [35]. The capacity to determine when, where, and how to access services is a critical element of NHL [35–38].

This subset operates at three hierarchical levels: macro (system-level understanding of healthcare structures, policies, and service availability), meso (analysing and using information to make healthcare decisions based on quality, cost, and convenience), and micro (direct interactions with healthcare providers, including communication and application of medical advice) [35]. Effective navigation involves expressing preferences and gathering information to participate in decision-making and health planning [35,38].

European healthcare systems, marked by complexity and a lack of transparency, pose considerable challenges to navigation, particularly for individuals with multiple health needs who often experience uncoordinated services [39,40]. Understanding how general and navigational health literacy interact is therefore critical for evaluating how individuals access and use health services [37,41].

Limited health literacy is consistently associated with higher healthcare utilisation and costs, poorer health outcomes, and greater financial burdens [42–47]. Additionally, it increases the likelihood of medical errors and communication difficulties with providers [11,48,49]. Individuals with inadequate health literacy face challenges in areas such as obtaining health insurance, accessing services, and understanding health information, which impedes their ability to manage their health effectively [47].

In Portugal, health literacy is notably low, with 44% of higher education students showing limited health literacy [50]. This issue is particularly pronounced in the Alentejo region, where 82.3% of students exhibited limited health literacy, with a mean score of 19.3 on a scale of 0–50 [25]. The Alentejo faces unique contextual challenges, including low tertiary education rates, high dropout rates, and ongoing depopulation, which exacerbate barriers to health literacy and healthcare access [19,51,52]. The Alentejo region, characterised by low population density and limited healthcare infrastructure, provides a critical context for understanding the interplay between health literacy and healthcare access [19,51,52]. Although health literacy challenges in rural Portugal exist, they reflect global patterns where limited health literacy exacerbates inequities in healthcare access, especially among socioeconomically disadvantaged groups [11,35,53–55].

Notwithstanding the challenges previously mentioned, to the best of our knowledge, there has been a paucity of research exploring the barriers and facilitators affecting health literacy in the region. The absence of research on the perceptions of this demographic hinders the formulation of effective policies and interventions. Despite the growing body of literature on health literacy, there is still a paucity of research that addresses its specificities among rural higher education students, who face challenges due to possible geographical and institutional barriers.

This study aimed to explore the perceptions of higher education students in Alentejo, Portugal, with regard to health literacy, access to and utilisation of healthcare. In addition, the study sought to identify barriers, facilitators and interventions with a view to informing strategies that promote equity and strengthen students’ capacities in order to sustainably improve health outcomes. By addressing these objectives, the findings will illuminate region-specific barriers and facilitators, providing evidence to guide strategies that empower students, improve health outcomes and ensure equitable access to healthcare. It is expected that the research will contribute to the promotion of health equity and the fostering of sustainable improvements in rural health systems.

## Materials and methods

### Study design and setting

An exploratory, descriptive, and qualitative study was conducted using focus groups [56] and content analysis techniques [57]. The Consolidated Criteria for Reporting Qualitative Research (COREQ) checklist guided the reporting of this study [57]. The study aimed to explore factors influencing health literacy, access, and utilisation of healthcare services among students from public higher education institutions in the Alentejo region, Portugal.

### Participant selection

The recruitment strategy aimed to reflect the diversity of higher education students in the Alentejo region, acknowledging that non-probability sampling may limit generalisability but ensures a focus on lived experiences [58]. Participants were recruited from all four public higher education institutions operating in the Alentejo region: the University of Évora, the Polytechnic Institute of Beja, the Polytechnic Institute of Portalegre, and the Polytechnic Institute of Santarém. These institutions collectively represent 100% of the public higher education sector in Alentejo, thereby ensuring comprehensive regional coverage and enhancing the contextual relevance of the findings. The inclusion of students from all public institutions in the region strengthens the representativeness of the sample within the specific geographical and institutional context under study.

No selection process was applied; all students who expressed interest and met the inclusion criteria were accepted to participate. To capture diverse perspectives, three focus groups were conducted with students in their first year, intermediate years, and final year, respectively. Recruitment was facilitated by Health Literacy National Network (HLNN) members and course coordinators, who distributed invitations via email. These invitations explained the study’s objectives and provided a link to the informed consent form. To ensure ethical compliance, the research team only accessed students’ contact information after their consent had been obtained through the coordinators.

Eligible participants met the following inclusion criteria: enrolment in higher education; proficiency in Portuguese; and voluntary willingness to participate. Additional efforts were made to include students from a range of academic disciplines and years of study, in order to enhance the representativeness of the sample within the regional context.

In line with established methodological guidance for focus group research [56,59,60], each group comprised between 4 and 12 participants. For each academic year (first, intermediate, and final), 24 students (six per institution) were invited to participate, anticipating possible attrition to maintain adequate group sizes. No incentives were offered for participation, in order to minimise response bias and preserve the voluntary nature of involvement.

### Setting

Focus groups were conducted via Zoom to enable students in geographically dispersed areas of the Alentejo region to participate. This approach ensured greater accessibility and inclusivity by overcoming logistical barriers such as limited transport infrastructure and long travel distances [61]. Furthermore, the online format offered a more flexible and time-efficient engagement method, accommodating the participants’ diverse schedules and academic commitments [61,62]. A total of 29 students participated in the study, with nine taking part in the first focus group, 10 in the second, and 10 in the third.

To minimise hierarchical influences, the research team prioritised an inclusive environment. Following the methodological guidelines of Krueger and Casey [56], each session included a moderator (J.R.), responsible for facilitating discussions, and an assistant moderator (R.P.), who observed, took notes, and ensured the sessions ran smoothly. Both moderators were introduced as neutral facilitators to mitigate perceived authority.

### Data collection

Data collection employed a semi-structured interview guide aligned with the study’s objectives. The following topics were addressed: factors that facilitate or hinder health literacy, factors that facilitate or limit access to health services, factors that facilitate or hinder the use of health services, and interventions to improve health literacy, access, and utilisation of healthcare services.

To ensure transparency and reproducibility, the complete interview guide is included as an appendix. Sociodemographic data, including age, gender, year of study, and academic institution, were collected via an online form prior to the sessions.

The focus groups were conducted in 11^th^, 13^th^, and 18^th^ December 2023, with each session lasting approximately 90 minutes. All sessions were recorded (audio and video) with participants’ prior consent.

### Data analysis

All focus group discussions were transcribed verbatim by the first author (J.R.) and independently reviewed by another author (J.P.) to ensure accuracy. The data were then analysed using content analysis techniques [63], comprising three phases. In the preliminary phase, researchers conducted an initial review of transcripts and observational notes to familiarise themselves with the data. During the exploration of the material phase, an inductive approach was employed to facilitate the coding and categorisation procedures. This approach enabled the emergence of themes directly from the data, without recourse to preconceived theoretical frameworks. The analysis was conducted in an open and iterative manner, with a view to ensuring that any emerging patterns were systematically captured. To ensure qualitative rigour, systematic comparisons were made between data segments in order to guarantee consistency and validity. The first and second authors engaged in a process of triangulation, whereby they cross-checked codes, discussed discrepancies, and sought to achieve consensus in order to minimise the potential for bias. The data set, which comprised focus groups, provided a robust foundation for inductive analysis. In the phase of treatment of results, inference and interpretation, categories and subcategories were refined iteratively, with support from discussions among the research team and validation against the objectives of the study to ensure relevance and coherence.

To illustrate the findings, anonymised context units were coded (e.g., “FG1, P3” for participant 3 from the first focus group). The point at which no new themes emerged after the third focus group was defined as data saturation, a concept that aligns with the qualitative research guidelines [58].

To ensure transparency and mitigate potential biases, the research team engaged in reflexive practices, critically examining their positionality and influence throughout the data collection and analysis process. Strategies such as triangulation, peer debriefing, and iterative consensus discussions among researchers were employed to enhance the credibility and dependability of the findings.

### Validity and rigor

The study employed several strategies to enhance validity and rigor [64]:

- Triangulation: Multiple researchers were involved in coding and analysis to reduce bias.
- Credibility: Expert consultations were conducted to validate the coding framework.
- Transferability: Detailed reporting ensures findings can be applied to similar contexts.
- Reflexivity: Researchers critically reflected on potential biases throughout the study, particularly regarding their roles in moderating the focus groups.

### Data processing tools

Data were managed and analysed using MAXQDA22 software, ensuring systematic organisation and traceability of coding decisions. The final categorisation (“Tree of Nodes”) was iteratively refined until all team members reached consensus. The MAXQDA22 software was utilised systematically to organise, code, and categorise data, enabling an iterative and transparent thematic analysis. Additional verbatim excerpts from participants were included to substantiate the themes, providing deeper insights into the qualitative narratives.

### Ethical considerations

This study was approved by the Ethics Committee of the University of Évora (Document number 22091). Written informed consent was obtained from all participants prior to data collection.

## Results

### Students’ characteristics

The participants in the focus groups were divided into three groups according to their academic year. The first focus group (FG1) comprised first-year students (n = 9; 31.0%), the second (FG2) middle-year students (n = 10; 34.5%), and the third (FG3) final-year students (n = 10; 34.5%). A total of 29 students participated in the study, with an age range of 18–25 years (mean age = 21.34 ± 2.87 years). The sample of the study consisted of 29 participants, including 14 (48.3%) males and 15 (51.7%) females. Participants were enrolled in various institutions, including the Polytechnic Institute of Beja (24.6%), the Polytechnic Institute of Portalegre (20.7%), the Polytechnic Institute of Santarém (27.6%), and the University of Évora (24.1%). Furthermore, 37.9% of participants were displaced, while 62.1% were not.

The participants were selected from a range of academic disciplines, comprising health-related courses (n = 9; 31.0%) and non-health-related courses (n = 20; 69.0%).

The findings are organised into three tables, each corresponding to a major thematic area derived from the inductive and content analyses of the focus group data: (i) health literacy, (ii) access and use of healthcare services in NHL, and (iii) interventions to improve health literacy.

### Health literacy

The focus group analysis identified key facilitators of health literacy, including access to digital resources, institutional support, and family networks. Conversely, several barriers were also highlighted, such as misinformation, limited critical health literacy skills, financial difficulties, insufficient health education, and the presence of emotional and social stigma. Collectively, these barriers undermined students’ capacity to seek, interpret, and effectively utilise healthcare services. Table 1 provides a detailed overview of the categories and subcategories derived from the coding process, outlining the thematic framework that guided the analysis.

**Table 1:** Thematic structure on health literacy among higher education students in Alentejo: Description of categories, sub-categories, themes, and student experiences related to health literacy, including digital access, information credibility, support networks, and structural challenges.

Category: Health Literacy						
Sub-category	Theme	Enumeration Unit	Codes (Units of Analysis)	Representative Quotations	Policy/Practice Implications	Count
Access to Digital Resources	Use of the internet to access validated health information sources (e.g., institutional websites, scientific databases)	Use of the internet to search for evidence-based health information	Online health searches; use of official websites; immediate access in urgent situations	“I think the internet is the best place (...) just search quickly and the information comes up right away.” (FG1, P6)	Develop targeted interventions to enhance critical digital health literacy skills among students. Incorporate modules into higher education curricula.	17
	Use of artificial intelligence (AI) for health-related inquiries (e.g., chatbot)	Use of AI tools for reliable health information	Use of AI-based platforms (e.g., ChatGPT) for immediate health-related guidance; perceived immediacy and practicality	“Nowadays with things like ChatGPT, you can get information faster.” (FG1, P6)	Establish guidelines and training for evaluating AI-generated health content. Promote responsible use of emerging technologies.	3
Information credibility	Exposure to health misinformation	Overload of inaccurate or non-validated content, causing confusion	Online health misinformation; difficulty in distinguishing credible from non-credible sources; misleading content	“There’s too much information… sometimes you can’t tell what’s true or false.” (FG2, P8)	Implement health communication campaigns focusing on media literacy and verification of online health content.	3
	Skepticism towards AI and online sources	Mistrust of digital health platforms and AI tools	Distrust in digital platforms; concern over source reliability; resistance to non-human input	“I don’t trust AI tools or websites... it’s hard to know who is behind the information.” (FG2, P8)	Strengthen trust in public digital health tools through certification, transparency, and endorsement by health authorities.	10
	Critical appraisal of health information	Skills in evaluating and interpreting health information	Ability to assess source credibility; filtering misleading titles; recognising clickbait	“Sometimes the titles are really eye-catching, but the actual content doesn’t match... you have to be critical.” (FG3, P3)	Integrate health information appraisal into university-wide literacy initiatives, including in non-health-related courses.	14
Support Networks	Family and peer support	Emotional and informational support from family and peers	Parental guidance; friends in health fields	“I usually call my mum first... but for actual health decisions, I talk to friends who study nursing.” (FG2, P5)	Strengthen peer-led health initiatives and incorporate family involvement in awareness campaigns.	5
	Professional health advice	Access to guidance from healthcare professionals	Informal consultations during internships; contact with lecturers in health fields	“Sometimes I ask a professor or a nurse I know. They can help without needing to go to the centre.” (FG2, P4)	Promote structured mentorships and supervised guidance during academic placements.	5
Institutional Educational Resources	Access to on-campus libraries and educational sessions	Use of university library and learning centres	Restricted library hours; desire for better access	“Our library closes too early... We’d use it more if it stayed open longer.” (FG3, P2)	Extend library opening hours and integrate health literacy sessions in curricular and extracurricular formats.	5
	Health-related educational sessions	Participation in health-oriented seminars, workshops, or lectures	Talks by student associations or lecturers	“The school organises several health-related projects. They help us become more informed.” (FG2, P4)	Encourage institutional partnerships with student groups to expand health literacy training.	12
Structural barriers	Limited formal/informal health education	Lack of access to structured health education	Curricular gaps; non-health students unprepared	“Those who are not from health degrees often don’t know where to go or what to do.” (FG2, P5)	Expand health literacy training beyond healthcare courses to all academic programmes.	12
	Financial barriers	Inability to afford healthcare, transportation, or educational sessions	Financial limitations affecting healthcare access, including inability to afford transport	“I had to skip a health workshop because I couldn’t afford the bus fare.” (FG3, P2)	Introduce subsidies or travel support for low-income students accessing health services.	2
Emotional and Stigmatising Factors	Stigma related to seeking care	Shame or embarrassment in accessing healthcare services	Embarrassment about sexual health topics; peer judgement	“I wouldn’t go to the health centre just for that... people would judge.” (FG1, P6)	Implement anonymised and youth-friendly services, especially for sexual and mental health.	5

### Access to digital resources

The ability to access and critically engage with digital resources has emerged as a key factor in developing health literacy among higher education students. The internet was identified as the primary source of health-related information across all focus groups (n = 17 coded segments). Students reported using online searches to manage minor symptoms and non-urgent concerns prior to seeking professional care. As one participant stated, “I usually do some online research to gain a better understanding of the symptoms before consulting a professional” (FG1, P3).

The use of generative artificial intelligence (AI) tools for preliminary health guidance was referenced in three coded segments (n = 3). These tools were valued for their speed and accessibility; however, participants also emphasised the need for critical evaluation. One participant observed, “It is unclear whether online artificial intelligence resources can be trusted to provide reliable health advice” (FG1, P1).

### Reliability of the information

Concerns about the reliability of digital health information were reported in 27 coded segments. These included references to misinformation (n = 3), scepticism towards AI tools (n = 10), and the need for critical evaluation (n = 14). Participants described difficulties in discerning credible sources due to the prevalence of contradictory content. As one participant stated, “The abundance of contradictory information on the Internet renders it challenging to ascertain the veracity of a given source” (FG2, P2).

The development of critical health literacy skills was consistently emphasised as essential for navigating digital environments. As one participant explained, “It is crucial to interpret the information we encounter online” (FG2, P4).

### Family, social and professional support network

Support from family members, peers, and healthcare professionals was identified as a key facilitator of health literacy (n = 10 coded segments). Family members were frequently consulted to validate online information (n = 5). One participant explained, “I contact my mother after consulting online sources to request her opinion” (FG2, P1).

Professional support, including both formal and informal consultations, was also cited (n = 5). Participants referenced services such as the national telehealth line (Saúde 24) as instrumental in guiding healthcare decisions. One participant noted, “On occasion, I contact Saúde 24 (a telehealth counselling service) to obtain advice prior to seeking emergency care” (FG1, P2). Another added, “Rapid consultations, even informal, with healthcare providers are crucial when uncertainty arises” (FG3, P1).

Despite the presence of familial and professional support, a lack of institutional mechanisms to assist students in navigating healthcare systems was repeatedly raised (n = 19 coded segments). As one participant observed, “There is a dearth of guidance available to students to facilitate informed decision-making regarding their health” (FG1, P5). A comparable concern was expressed by another participant: “There is a dearth of formal assistance, which leaves us to navigate these matters independently” (FG3, P3).

### Institutional resources

Institutional resources were identified as facilitators of health literacy (n = 17 coded segments). Participants referred to access to university libraries, health-related educational activities, and on-campus healthcare professionals. Libraries were valued for providing reliable sources of information and for enabling students to verify online content (n = 5). One participant stated, “Access to trusted books in the library enables me to verify the information I find online” (FG3, P3). Another added, “Someone who is there, at school, a health professional at school, a nurse, helps a lot, not just to answer questions” (FG1, P3).

Workshops and webinars were also mentioned as important mechanisms for promoting health literacy, particularly in areas such as mental health, sexual health, and emergency care (n = 12 coded segments). However, participants also noted their limited availability. One student shared, “We also had access to the webinars, it was very good,” and, “I believe this (webinars) is highly important” (FG3, P6).

### Structural challenges

Structural barriers to health literacy were consistently reported. The absence of formal health literacy education in higher education curricula was noted in 14 coded segments. Participants expressed a clear need for structured instruction, highlighting its relevance to autonomy and wellbeing. One participant commented, “There is a lack of awareness regarding the significance of health literacy at the higher education level” (FG1, P4). Another concurred, stating, “Health education is a subject that should be included, particularly at the university level” (FG3, P3).

Financial constraints were also cited as limiting participation in health-related educational opportunities (n = 12 coded segments). Students reported that the cost of attending workshops or seminars often precluded access. As one participant explained, “On occasion, financial constraints prevent students from attending health workshops, unless they are provided at no cost” (FG2, P1).

### Emotional and stigmatising factors

Emotional and societal barriers were identified as obstacles to achieving health literacy, particularly with regard to mental and sexual health. A total of five coded segments (n = 5) reflected participants’ reluctance to seek help due to shame or fear of judgement. One participant noted, “It’s embarrassing to seek assistance for sexual health concerns (...) in our sexuality, sexual orientation, sexual relationships, we have doubts, fears and sometimes we don’t ask because of shame and fear of being made fun of” (FG2, P4). Another added, “Discussing mental health with family or friends is often challenging, which can impede the ability to seek and access the necessary support” (FG1, P2).

### Access to and utilisation of healthcare services in navigational health literacy

Thematic analysis revealed that students’ ability to access and utilise healthcare services was shaped by a dynamic interplay of enabling and constraining factors. These were categorised into four subdomains: (1) family and institutional support networks; (2) the responsiveness and organisation of health services; (3) economic capacity and logistical resources; and (4) knowledge of access procedures and health-related rights. These domains closely align with the core dimensions of navigational health literacy—namely, the ability to access, understand, evaluate, and apply health-related information to obtain appropriate care, in the right place, at the right time. Table 2 explores these domains in detail, illustrating both facilitators and barriers that influence students’ healthcare experiences, particularly among those enrolled in non-health-related programmes.

**Table 2:** Thematic analysis of structural, institutional, and informational factors influencing students’ access to and use of healthcare services.

Category: Access to and utilisation of healthcare services in NHL						
Sub-category	Theme	Enumeration Unit	Codes (Units of Analysis)	Representative Quotations	Policy/Practice Implications	Count
Social and Institutional Support	Family and peer support	Dependence on family support for navigating healthcare	Parental advice; lack of local support networks	“My parents aren’t in Portugal… if I get sick, I just go online or call Saúde 24.” (FG2, P5)	Promote digital and peer-navigation tools for students living away from home.	7
	Institutional support gaps	Lack of healthcare-related guidance and support within educational institution	Absence of health offices; students unaware of available services	“We don’t have a doctor or nurse on campus... there’s nowhere to go if something happens.” (FG3, P9)	Develop institutional health response protocols and campus-based health access points.	19
Health System Responsiveness	Emergency services access	Free, universal access to emergency healthcare services	First recourse in absence of primary care; perceived as more reliable	“There’s always an open door — that’s the emergency department.” (FG1, P1)	Enhance triage literacy and promote alternatives to emergency departments for non-urgent needs.	12
	Waiting times	Delays and long waiting periods in accessing healthcare services	Delayed appointments; missed consultations due to scheduling issues	“By the time I got an appointment, I was already better, so I never went.” (FG3, P4)	Implement fast-track systems or student-specific appointment slots.	3
	Navigation challenges	Absence of guidance within the healthcare system	Difficulty identifying appropriate points of access; lack of support	“I didn’t know where to go, so I ended up at emergency for something simple.” (FG2, P6)	Disseminate system navigation guides for students at enrolment.	10
	Educational exposure to healthcare	Attending a course in the health field	Knowledge of healthcare pathways due to course content	“As nursing students, we’re taught about the system, but others are lost.” (FG2, P5)	Extend basic health system orientation to all students, not just health majors.	1
	Difficulty in scheduling appointments	Logistical and administrative barriers to securing timely medical consultations	Unanswered calls; delays; email issues; scheduling conflicts with classes	“I couldn’t get through on the phone… I missed three appointments.” (FG3, P7)	Introduce online booking systems with student-adapted scheduling.	30
	Lack of system registration	Absence of health card, assigned family doctor, or registration in local healthcare units near the place of study (displaced students)	Not accepted at local units; referred to emergency services	“They wouldn’t see me because I’m not registered here.” (FG3, P9)	Implement mobile registration teams or facilitate temporary enrolment for displaced students.	14
Knowledge of Access Procedures and Health Rights	Understanding of healthcare access and entitlements	Lack of formal mechanisms to support understanding of healthcare access pathways	Students unaware of entitlements; lack of institutional support	“We don’t know what we have a right to... we just guess or Google it.” (FG3, P2)	Include healthcare rights and system information in orientation materials.	6
	Navigating the healthcare system	Limited knowledge of how to access and navigate healthcare services	Confusion regarding procedures, locations, and responsibilities	“I didn’t even know we had a psychologist on campus.” (FG2, P4)	Establish clear communication strategies and visual system maps across campuses.	13
Economic and Logistical Resources	Health insurance coverage	Availability of insurance for transport and healthcare expenses	Some students with private plans or parental coverage	“With my parents’ insurance I go private, it’s faster.” (FG3, P8)	Provide guidance on public/private integration and financial literacy in health planning.	4
	Financial constraints	Insufficient financial resources to cover medical costs and transport	Missed appointments; inability to afford public transport	“I skipped a doctor’s visit because I couldn’t pay for the bus.” (FG2, P6)	Offer travel vouchers or institutional co-funding for students in need.	11

### Family, social and institutional support network

Family support was referenced in seven coded segments (n = 7), where participants described relying on relatives for guidance in recognising when and how to seek care. One student noted, “My family provides me with guidance on how to access appropriate services when I am unwell” (FG3, P2). Another added, “My family provides guidance on when it is appropriate to seek healthcare” (FG2, P3).

By contrast, institutional support was viewed as insufficient, with 19 coded segments (n = 19) highlighting the lack of structured mechanisms to help students—especially those living away from home—navigate healthcare systems. One participant stated, “The university’s additional support in the field of health helps to speed up access to health services, but there is none of that” (FG3, P1). Another stressed, “It is challenging to access healthcare when one is residing at a distance from one’s place of origin and lacks the support of family members in the vicinity” (FG3, P1).

### Response capacity and organisation of health services

While participants acknowledged the importance of accessible emergency services, systemic inefficiencies were raised in 33 coded segments (n = 33), including long waiting times, difficulties in scheduling, and care delays. One participant remarked, “On occasion, the waiting period is unacceptably lengthy” (FG3, P2), while another added, “The waiting periods and appointment scheduling difficulties within the system are unacceptable” (FG2, P2). These delays were not limited to scheduling: “Delays are not exclusive to the scheduling of appointments but also pertain to the waiting period at the clinic” (FG3, P5).

Participants also expressed difficulty in navigating the healthcare system due to a lack of institutional guidance. As one student noted, “There is a lack of guidance when it comes to navigating the healthcare system” (FG1, P4), and another added, “There is a clear need for greater institutional support in order to facilitate the navigation of healthcare services” (FG2, P3).

Participants with prior exposure to health-related education reported greater ease in navigating the system. One student explained, “The acquisition of knowledge from health-related courses or workshops facilitates the timely and appropriate utilisation of healthcare services” (FG1, P5). Awareness of available resources, such as telehealth services and local health centres, was also considered helpful: “Awareness of the services available, such as emergency telephone numbers or local health centres (Health Centres of the National Health Service), facilitates access to care” (FG3, P3).

Continuity of care was described as a facilitator by participants who had consistent access to a general practitioner. As one noted, “The presence of a regular family doctor facilitates the follow-up of treatment when required” (FG1, P3).

### Knowledge of access procedures and health rights

Understanding healthcare access procedures and entitlements was described as a key facilitator in nine coded segments (n = 9). Participants emphasised that such knowledge supports timely and appropriate use of healthcare services. One student remarked, “Having a clear understanding of the relevant procedures and one’s entitlements facilitates a more streamlined process” (FG2, P4). Another highlighted the benefit of academic exposure to health fields: “Being a student in a health-related field is advantageous as it provides an understanding of the procedures for accessing healthcare” (FG1, P3).

Nonetheless, displaced students described barriers to continuity of care, particularly in managing chronic conditions. These barriers stemmed from their exclusion from local health centre registries, which restricted access to routine follow-up care. As a result, they often resorted to emergency services. One participant explained, “The distance from home and my usual healthcare providers makes it more challenging to manage my health, particularly when I require follow-up appointments for my chronic disease” (FG3, P3).

### Economic capacity, financial support and logistical resources

Economic and logistical capacity were reported as pivotal factors influencing healthcare access (n = 11 coded segments). Participants cited financial resources, including health insurance, as key facilitators. One student stated, “Health insurance is of considerable assistance; otherwise, accessing private care would be unfeasible” (FG2, P5).

In parallel, transportation-related costs were consistently referenced as barriers. Students described how financial constraints impeded their ability to travel to healthcare facilities. One participant explained, “If individuals lack the financial means to travel, they are unable to access healthcare (...) The availability of financial resources for transportation and medical consultations facilitates the acquisition of necessary healthcare services” (FG1, P1). Another echoed, “In the absence of financial resources for transportation, it is exceedingly challenging to reach the clinic or hospital” (FG2, P1).

### Interventions to improve HL and NHL

The study identified a series of targeted interventions designed to enhance health literacy (HL) and navigational health literacy (NHL) among higher education students in the Alentejo region. These interventions, identified across 21 coded references, reflect a structured and multidimensional strategy comprising five key components: (1) thematic workshops and health education sessions; (2) on-campus access to clinical support; (3) development of student-centred digital tools; (4) mechanisms for continuous evaluation and monitoring; and (5) institutional collaboration with student associations and local healthcare providers. Table 3 presents these components in detail, including illustrative quotations, policy and practice implications, and the frequency with which each theme was mentioned, offering practical strategies to address the barriers identified and strengthen student health support systems.

**Table 3:** Recommendations derived from student suggestions and lived experiences, focusing on educational activities, institutional support, digital tools, and collaborative strategies.

Category: Interventions to Improve HL and NHL						
Sub-category	Theme	Enumeration Unit	Codes (Units of Analysis)	Representative Quotations	Policy/Practice Implications	Count
Educational Activities	Health promotion Workshops	Participation in training on mental health, reproductive health, and emergency response	Webinars; mental health workshops; CPR training	“There was a webinar about anxiety attacks… it helped a lot.” (FG2, P6) “We have Basic Life Support workshops organised by the student association, but they often clash with classes.” (FG3, P3)	Allocate dedicated time slots for health workshops. Integrate these into academic timetables.	3
On-campus Services	On-campus healthcare professionals	Availability of health professionals for consultations, especially sexual health	Presence of psychologists; lack of doctors/nurses on campus; care deferral to emergency department	“We have a psychologist but no nurse or doctor. We’re sent to the emergency department for minor issues.” (FG3, P9)	Establish multi-professional health offices on campus for basic and preventive care.	2
	Support for displaced students	Personalised health orientation for students studying away from home	Difficulty registering with local GPs; lack of institutional guidance	“I was turned away because I wasn’t registered locally. A campus nurse could’ve helped.” (FG3, P9)	Provide health navigators or institutional advisors for non-local students.	2
Digital Health Tools	Student-targeted mobile applications	Development of apps to support health system navigation and student assistance	Absence of centralised digital platforms; fragmented access to information	“We don’t really have an app to help us find what services are available or how to book.” (FG3, P2)	Create student-centred apps with health service mapping, booking, and education features.	1
	Personalised health guidance	Tailored interventions based on students’ specific health needs	Suggestions for personal consultations or digital triage tools	“We need someone to help guide us... not just general advice.” (FG1, P5)	Implement personalised digital or in-person triage and referral systems.	2
Evaluation and Monitoring	Mechanisms for assessing health literacy	Involvement of health professionals (e.g., nurses) in monitoring and improving health literacy	Ideas for institutionalised literacy evaluation; follow-up	“Maybe a nurse could help us understand how well we know things or guide us better.” (FG2, P6)	Develop routine HL assessment protocols within student health services.	4
	Monitoring engagement with services	Tracking attendance in workshops and utilisation of campus health services	Lack of usage metrics; invisibility of participation outcomes	“We attend, but there’s no follow-up… not even a certificate or feedback.” (FG3, P3)	Track and evaluate student participation to refine health promotion strategies.	2
Collaborative Strategies	Partnerships with Student Associations	Joint health promotion initiatives with student organisations	Student groups as drivers of health education activities	“Many initiatives are led by student unions, but they need more support from the institution.” (FG2, P4)	Strengthen formal institutional collaboration with student-led health initiatives.	2
	Collaboration with Local Healthcare Providers	Formal agreements with local health services	Desire for dedicated student slots in local clinics	“It would help if there were reserved appointments for students.” (FG2, P5)	Establish “green lines” or reserved appointment quotas with nearby healthcare providers.	2
	Priority access protocols for students	Special referral mechanisms (e.g., “green line”) to expedite student access to care	Barriers due to lack of local registration; delayed care	“We should have a way to get access faster as students without clogging emergency.” (FG2, P6)	Develop streamlined referral pathways for higher education students, especially displaced ones.	2

### Workshops and training programmes

Workshops and thematic training sessions were recognised as essential components of the intervention strategy (n = 12 coded segments). Participants recommended that these sessions address critical health topics, including emergency preparedness, mental health, and reproductive health. A blended learning model (combining online and in-person formats) was proposed to maximise accessibility and engagement.

Interactive, scenario-based pedagogical approaches were strongly endorsed to enhance knowledge retention and practical application. One participant articulated this need, stating:

“There should be compulsory preparatory training that includes what to do in the event of earthquakes, road accidents, avoiding pregnancy, avoiding violence, help for depression and so on, so that we know where to go when we have a problem, or think we do, as there’s nothing, we either go to the emergency room or we don’t go anywhere else, it’s only when we’re really bad that we ask for help” (FG1, P9).

Three participants (n = 3) recommended the periodic organisation of thematic workshops and the integration of tailored educational modules into the academic curriculum to ensure sustained and comprehensive coverage of essential health topics.

### On-campus support provided by nurses or doctors for HL and NHL

Systemic inefficiencies in the healthcare system were highlighted in 33 coded segments, with students reporting prolonged waiting times and delays in appointment scheduling. These barriers were perceived as particularly detrimental to timely access to care.

It was referred by the participants (n = 2) preference for the establishment of on-campus healthcare support services, specifically advocating for the presence of a nurse or general practitioner. This intervention was viewed as especially beneficial for geographically displaced students, who often face additional logistical and administrative barriers. As one participant noted, “A nurse or doctor (at University campus) would be especially helpful for displaced students” (FG3, P10).

The integration of on-campus clinical support was considered a potentially effective strategy to reduce reliance on emergency services, promote early engagement with preventive care, and improve continuity of care for students with limited access to local health systems.

### Mobile application development

The development of a student-centred mobile application to support navigation of health services was proposed in one coded segment (n = 1), indicating an emergent but limited theme. The application was envisioned as a digital tool to enhance access to reliable health information and facilitate informed decision-making. As one participant suggested, “We should have a course, online, something, so we know what to do and where to go. Something done on a mobile phone, like a student app” (FG1, P5).

### Assessment and evaluation mechanisms

The importance of continuous monitoring and evaluation of health literacy interventions was emphasised in six coded segments (n = 6), with specific reference to the role of dedicated health professionals (n = 4) and systematic tracking mechanisms (n = 2). Participants highlighted the need for structured feedback loops to assess the effectiveness of interventions, clarify doubts, and measure behavioural or knowledge-based outcomes over time.

One participant remarked, “If we had a professional who could help us in this respect (clarify doubts), I think it would be fantastic, as it helps, I think it would help a lot of young people” (FG2, P3). These findings underscore the necessity of robust evaluation frameworks to ensure the relevance, responsiveness, and long-term impact of health literacy initiatives.

### Collaborations with student associations and local health providers

Structured partnerships between student associations and local healthcare providers were proposed in six coded segments (n = 6), indicating a recurring theme across focus groups. Participants viewed such collaborations as essential for enhancing student engagement, improving access to services, and tailoring interventions to the specific needs of the academic community.

One participant illustrated this point by stating, “If the Portuguese Institute for Sport and Youth were to form a partnership with the local health centres, it would be possible to arrange a number of appointments a day specifically for students” (FG3, P5). These partnerships were seen as a mechanism to institutionalise support structures and foster a more integrated and sustainable approach to student health and wellbeing.

## Discussion

This study examined the perceptions of higher education students in Alentejo, Portugal, regarding health literacy and access to healthcare, and identified key barriers, facilitators and student-driven recommendations. The findings highlight the need for a multifaceted approach to health literacy that involves autonomous learning, institutional support, and engagement with trusted networks. Students face challenges such as economic constraints, stigma, and structural obstacles, but family support, institutional resources, and peer networks can help address these issues.

The 29 participants represented diverse academic backgrounds and demographics, enriching perspectives on health literacy and navigation. The diversity of the sample is indicative of a range of socio-demographic backgrounds and academic trajectories, which is essential to understanding how HL and NHL are influenced by these factors. Students from different academic disciplines may approach health-related issues with different knowledge and skills, particularly when it comes to navigating health systems [65]. The inclusion of both health-related and non-health-related students enables a more nuanced exploration of HL and NHL, given that health-related students have higher levels of health literacy [12,25,36].

The analysis is framed within the HL Model and the NHL framework, which guided the exploration of students’ capacity to access, understand, and apply health information [1,3,35,38,43,66]. These models underscore the dynamic interaction between functional, critical, and navigational HL [3,43,66]. The study highlights that HL is not just about the ability to obtain information, but also the critical skills needed to evaluate and apply this knowledge effectively in health decision-making. The HL model emphasizes the importance of functional and critical health literacy as key determinants for managing health and navigating healthcare systems [5,43,66]. The findings demonstrate that digital tools, particularly the internet, play a pivotal role in accessing health information. However, challenges surrounding the evaluation of the credibility of online resources underscore the necessity for enhanced eHealth literacy (the capacity to critically assess online health information), which constitutes a component of critical health literacy.

### Health literacy

This study highlights the dual importance of functional and critical health literacy as key determinants of individuals’ ability to manage their health and navigate complex health systems. HL, as conceptualised in the HL model, goes beyond the ability to access and understand health information; it requires critical skills to evaluate and effectively apply knowledge to make informed health decisions [5]. The results align with the view of health literacy as a global public priority, vital for achieving the Sustainable Development Goals and the 2030 Agenda’s ambitions, and controlling noncommunicable diseases [67,68].

Digital tools, particularly the internet, play a key role in health literacy. However, students’ ability to critically evaluate online information is lacking, which highlights the urgent need to improve eHealth literacy. This statement aligns with other Portuguese scientific studies on health literacy among higher education students, emphasizing the need to empower students to access reliable health resources and foster a culture of health literacy that can positively impact their well-being both during their academic journey and beyond graduation [50,54,69]. Such skills are essential for navigating modern healthcare environments, where digital misinformation poses a serious threat to informed health behaviours [70–72].

Students’ perspectives are consistent with evidence on the functional aspect of health literacy, where individuals seek information to address minor health concerns [66]. The increasing reliance on digital resources further emphasises their role in contemporary health literacy [5,73]. Nevertheless, digital barriers persist, as participants frequently reported challenges in assessing the credibility of online information. Students also demonstrated a lack of awareness of the risks associated with misinformation, which confirms the findings of studies that highlight the difficulties many people face when evaluating online health resources [72,74–76].

These findings highlight the critical dimension of eHealth literacy, which enables individuals to distinguish credible health information from misleading content [77]. They also highlight the necessity of promoting comprehensive health literacy, particularly in digital contexts, to empower individuals to make informed health decisions [69,78,79]. This perspective fits in with critical health literacy, which emphasizes the analytical and application skills needed to effectively integrate health information into real-life contexts [66].

### Navigational health literacy: Access to and use healthcare services

From the perspective of higher education students in the Alentejo region, access to and use of health services requires NHL, which implies the ability to understand information and navigate health systems effectively. The findings revealed that financial constraints and the lack of culturally sensitive care act as major barriers to accessing these services. Furthermore, the importance of family support and institutional resources, such as on-campus health services and workshops, was emphasised as a key factor in addressing these challenges.

Financial constraints served to compound the issue of limited access, thereby impeding participants’ ability to obtain essential healthcare services. Furthermore, the lack of culturally sensitive care and the perceived stigma surrounding mental health services were identified as additional barriers that discouraged healthcare engagement [77,79]. Furthermore, institutional challenges, including the limited availability of on-campus health services and a lack of awareness about existing resources, served to compound these obstacles [66]. These considerations are in accordance with the NHL framework, which highlights the necessity to furnish students with the essential skills and confidence to utilise healthcare resources. This is particularly pertinent in contexts characterised by limited resources, such as rural areas, a situation that is exemplified by the Alentejo region.

The role of family support in facilitating navigation of the healthcare system was identified as a key facilitator factor. It was a common practice for participants to seek advice from family members in order to ascertain the gravity of their health issues and determine the most appropriate course of action. This highlights the fundamental role of personal networks in facilitating health navigation [38,40,80]. Similarly, institutional support was considered to be a fundamental factor in enhancing access to healthcare services. On-campus health services and educational workshops were identified as particularly valuable resources, with participants emphasising the necessity for higher education institutions to enhance health-related resources, particularly for students residing away from home. It is therefore imperative that such institutional support be expanded in order to address the gaps in healthcare access that currently exist and to foster effective health navigation.

### Health literacy interventions

For higher education students in Alentejo, targeted interventions should focus on enhancing eHealth literacy, critical thinking, and curricular integration. Institutions must provide verified digital resources, expand on-campus services, and actively reduce stigma, especially in mental and sexual health [79]. HL and patient participation are fundamental for sustainable development and the provision of effective healthcare services in the future [24].

This study emphasises the imperative for the development of bespoke strategies to enhance HL and facilitate improved healthcare accessibility. It is recommended that interventions focus on developing advanced eHealth literacy by equipping students with research and analytical skills, thus enabling them to engage with health information in a critical manner and make informed decisions. The available evidence indicates that formal HL education has a positive effect on the adoption of adaptive health behaviours and on health outcomes [2,5,81].

It is recommended that HL initiatives commence at an early age and continue throughout the life course, addressing the diverse demographic needs of the population. This is of particular importance in regions such as Alentejo, where population ageing and low population density serve to exacerbate existing healthcare disparities [25]. The implementation of tailored approaches can serve to mitigate these inequities and ensure equitable access to care [25,31,82,83].

The deployment of digital tools within the university context offers considerable potential for the advancement of HL [31,74,76,84]. It is the responsibility of educational institutions to ensure that students have access to authenticated online platforms and to provide eHealth literacy workshops, thus equipping them with the ability to discern reliable online resources. Such workshops have the potential to foster informed health management behaviours, thereby addressing an important gap in students’ HL [50,53,69,80]. Modules addressing the evaluation of digital health resources and the combating of misinformation are particularly relevant in the context of the digital age. They have the potential to play a pivotal role in fostering informed health management behaviours [13,53,69,80].

In a similar vein, it is imperative to fortify the institutional framework. This may prove advantageous in the extension of healthcare services on campus, the enhancement of awareness regarding existing resources, and the implementation of peer mentoring programmes. These measures are designed to assist students who may lack experience in navigating healthcare systems [13,80,83].

For some students, the availability of financial support can be a determining factor in whether or not they are able to access healthcare services. It is of equal importance to address the financial obstacles that impede access to healthcare. The provision of subsidised or free healthcare services can serve to alleviate economic constraints, thus ensuring that all students, regardless of their socio-economic background, have access to the healthcare they need [81]. The availability of financial support can often influence whether students seek healthcare services or forgo them due to cost concerns [5,25,50].

It is of the utmost importance to reduce stigma and enhance cultural sensitivity within healthcare services in order to encourage diverse student populations to engage with available resources [79,83,85,86]. The implementation of destigmatisation campaigns focusing on mental and sexual health, in conjunction with the provision of culturally tailored care, can facilitate the creation of inclusive environments that support student well-being [79,87]. These approaches are of particular importance for marginalised by society or underrepresented groups [7,77,79,86].

Although region-specific data remains limited, national studies highlight the critical need for targeted interventions to address the persistence of mental health stigma among Portuguese university students, particularly in underserved regions such as Alentejo, where structural and cultural barriers may further inhibit access to mental and sexual health services [88,89].

A comprehensive intervention to enhance HL and accessibility among higher education students in Alentejo must encompass culturally tailored educational programmes that address both functional and critical health literacy. It is recommended that such programmes place an emphasis on the evaluation and application of health information, while addressing the specific challenges faced in this region, including rural healthcare disparities and the distinctive sociodemographic context of Alentejo. Moreover, interventions should prioritise the provision of affordable healthcare services, the establishment of reliable digital platforms, the reduction of stigma surrounding mental health through targeted campaigns, and the reinforcement of institutional and community support structures to guarantee equitable and sustainable healthcare access. These action points are consistent with the scientific evidence in this regard [80,83,50].

To improve sexual and mental health outcomes, it is essential to implement targeted interventions that are culturally sensitive and grounded in evidence-based practices. Such programmes should address a number of key issues, including contraception, the prevention of sexually transmitted infections, the importance of consent, the promotion of healthy relationships, stress management and the enhancement of mental well-being. Furthermore, it is of the utmost importance to guarantee access to confidential, inclusive, and student-friendly healthcare and counselling services [36,37,90].

These results are in line with those of other international studies, which emphasise the fundamental role of HL in navigating health systems [1,34,35,38]. Similar interventions in rural and underserved populations around the world have demonstrated the effectiveness of tailored health literacy programmes in improving health outcomes and service utilisation [91–93]. Comparative analyses revealed the need for context-specific adaptations to address the distinctive barriers encountered by higher education students in Alentejo.

### Strengths and limitations of the study

This qualitative study provided a nuanced understanding of the lived experiences and perceptions of health literacy (HL) among higher education students. Including participants from different academic years enabled us to capture the evolving challenges and facilitators of HL. Applying the HL Model and NHL framework provided a robust theoretical basis for analysing students’ healthcare decision-making processes.

The group dynamics of focus group discussions may have introduced a social desirability bias, particularly given the sensitive nature of the topics explored, such as mental health, financial hardship and healthcare-seeking behaviours [61,62]. Despite efforts to cultivate an open and non-judgemental atmosphere, participants may have tailored their responses to align with perceived group norms or expectations. Although confidentiality and voluntary participation were emphasised throughout the process, this limitation highlights the need for caution when interpreting self-reported behaviours and attitudes. To mitigate such bias, future research could adopt a mixed-methods approach, incorporating individual interviews or anonymous surveys to triangulate data and enhance the credibility of the findings.

### Implications for public health practice and future research

The findings highlight the urgent need for integrated, context-sensitive strategies to improve HL among higher education students in rural areas. Higher education institutions must incorporate HL education into their curricula and develop peer-led initiatives that leverage existing social networks to foster environments conducive to autonomous and critical health learning. Effective implementation requires close collaboration between universities, healthcare providers and policymakers to comprehensively address systemic barriers.

Public health campaigns should adopt a more culturally sensitive approach to reduce stigma, particularly with regard to mental health and sexual health. These issues remain major impediments to care in rural populations. Simultaneously, expanding access to subsidised healthcare services and verified digital health resources is crucial to ensuring equitable utilisation of healthcare services.

Future research must prioritise evaluating the effectiveness of HL interventions, particularly in resource-limited rural settings. Longitudinal studies are essential for tracking changes in HL and assessing the sustained impact of interventions on health behaviours and outcomes. Investigating the integration of HL education into higher education curricula will clarify its role in empowering students to navigate complex healthcare systems effectively. Furthermore, research into tailored health services for students will inform the design of more effective HL promotion strategies.

Prioritising the development of equitable, context-specific care models is essential to reducing healthcare disparities and enhancing access. Institutions should actively partner with local healthcare providers to raise awareness of available resources and mitigate the logistical and financial challenges faced by students. These efforts must prioritise the creation of inclusive, culturally sensitive and accessible healthcare environments that encourage student engagement.

In practice, nurses play a pivotal role in health education, fostering supportive environments that encourage students to take a proactive approach to health management. The application of theoretical frameworks such as the Health Literacy Model and the Navigational Health Literacy Framework guides the design and implementation of effective, evidence-based interventions that can bridge existing gaps in healthcare access and literacy.

## Conclusion

This study emphasises the vital function of HL in enabling higher education students in the Alentejo region, particularly those in rural areas characterised by geographical dispersion and ageing populations, to navigate healthcare systems. While digital tools and social networks have the potential to improve access, persistent challenges such as misinformation, financial limitations and social stigma continue to hinder informed health decision-making.

The findings emphasise the importance of the HL and NHL models, particularly with regard to functional and critical HL. While functional HL enables basic engagement with healthcare services, critical HL empowers students to evaluate complex information and make autonomous decisions. Closing the gaps in eHealth literacy and tackling misinformation are essential to this empowerment process.

Integrating HL into higher education curricula is a strategic imperative. A comprehensive approach combining institutional support (e.g., workshops, on-campus healthcare and nursing-led interventions) with credible digital resources and peer networks can foster student resilience and close existing literacy gaps.

Higher education institutions play a pivotal role in promoting equitable and sustainable access to healthcare. By addressing regional healthcare challenges and equipping students with the skills to engage critically with health information, these institutions can significantly improve student well-being. Nursing-led initiatives, in particular, are a valuable way of strengthening health literacy among students and within the broader community, supporting the development of more resilient and sustainable healthcare practices.
